# Drought Eliminates the Difference in Root Trait Plasticity and Mycorrhizal Responsiveness of Two Semiarid Grassland Species with Contrasting Root System

**DOI:** 10.3390/ijms241210262

**Published:** 2023-06-17

**Authors:** Dongdong Duan, Xiaoxuan Feng, Nana Wu, Zhen Tian, Xin Dong, Huining Liu, Zhibiao Nan, Tao Chen

**Affiliations:** 1 State Key Laboratory of Herbage Improvement and Grassland Agro-Ecosystems, Center for Grassland Microbiome, College of Pastoral Agricultural Science and Technology, Lanzhou University, Lanzhou 730000, China; duandd17@lzu.edu.cn (D.D.);; 2 Sichuan Zoige Alpine Wetland Ecosystem National Observation and Research Station, Institute of Qinghai-Tibetan Plateau, Southwest Minzu University, Chengdu 610041, China

**Keywords:** arbuscular mycorrhizal fungi, climate change, drought, nutrient foraging, root system architecture, root traits

## Abstract

Root traits and arbuscular mycorrhizal (AM) fungi are important in determining the access of plants to soil resources. However, whether plants with different root systems (i.e., taproot vs. fibrous-root) exhibit different root trait plasticity and mycorrhizal responsiveness under drought remains largely unexplored. Tap-rooted *Lespedeza davurica* and fibrous-rooted *Stipa bungeana* were grown in monocultures in sterilized and live soils, followed by a drought treatment. Biomass, root traits, root colonization by AM fungi, and nutrient availability were evaluated. Drought decreased biomass and root diameter but increased the root:shoot ratio (RSR), specific root length (SRL), soil NO_3_^−^-N, and available P for the two species. Under control and drought conditions, soil sterilization significantly increased the RSR, SRL, and soil NO_3_^−^-N for *L. davurica*, but this only occurs under drought condition for *S. bungeana*. Soil sterilization significantly reduced AM fungal root colonization of both species, but drought significantly increased it in live soil. In water-abundant conditions, tap-rooted *L. davurica* may depend more on AM fungi than fibrous-rooted *S. bungeana*; however, under drought conditions, AM fungi are of equal importance in favoring both plant species to forage soil resources. These findings provide new insights for understanding the resource utilization strategies under climate change.

## 1. Introduction

Drought is a serious problem in most parts of the world, which causes strong and widespread impact on plants, threatening biodiversity, productivity, and stability of terrestrial ecosystems [1,2,3,4,5]. This is particularly the case for arid and semiarid grasslands because of their vulnerability to climatic changes [6,7]. Drought effects on plants are closely associated with the response of root traits, including root biomass allocation, specific root length (SRL), root diameter (RD), and root tissue density (RTD) and their function to water limitation, leading to altered plant nutrient uptake [8,9,10]. Thus, characterizing the plasticity of root traits to changing water availability is crucial for understanding the resource utilization strategies and performance of plant species in arid and semiarid grasslands under ongoing climate change.

However, the direction and magnitude of root trait plasticity in response to drought have been under debate [11,12,13,14]. Some studies have emphasized the resource economics hypothesis reporting that plant species exhibit conservative root traits (e.g., larger RD, lower SRL, and lower tissue nitrogen content) in response to drought, ensuring their survival under water-stressed conditions [15,16,17,18]. In contrast, other studies report that plant species would produce thinner roots with high SRL under drought conditions, supporting the functional equilibrium hypothesis that plants would be more acquisitive under conditions of lower resource availability [19,20]. A recent study found that grasses with a fibrous root system showed a more pronounced increase in RD and decrease in SRL than forbs or legumes with taproot systems under drought conditions [12]. Similarly, a study revealed that with the increase in aridity index, the root length in fibrous-rooted C_3_ species is greatly reduced compared to tap-rooted C_3_ species [21].

These contrasting patterns in root trait responses to drought potentially relate to the type of root system. There are two types of plant root systems: the taproot system characterized by a taproot and coarse lateral roots and the fibrous root system with numerous fine roots [22,23]. The root system architecture determines the ability of plants to access soil, water, and nutrients and, thus, influences plant growth [24,25,26]. Generally, plant species with a fine root architecture depend more on their root morphology; thus, they have a greater SRL to enhance water and nutrient acquisition from the soil [27,28,29,30,31]. Conversely, plants with a coarse root architecture tend to rely on greater growth benefit from arbuscular mycorrhizal (AM) fungi [32,33,34,35,36] as AM fungal hyphae can bypass water depletion zones around plant roots, allowing efficient exploration and acquisition of soil water and nutrients [37,38,39,40]. Although several studies have reported that plant growth dependence on mycorrhizal fungi is closely associated with their root system architecture [28,33,41,42,43], the extent to which this association is modified by drought remains largely unexplored.

In Northwest China, a vast area is occupied by arid and semiarid grasslands, which are sensitive to increasing anthropogenic disturbance and recurring drought events [6,14]. *Lespedeza davurica* (Laxm.) Schindl. (Fabaceae) and *Stipa bungeana* Trin. (Poaceae) are two dominant perennial species with important ecological and economic values in Northwest China [44,45]. They play a critical role in maintaining grassland productivity and reducing soil and water loss due to their high forage production, nutritional value, and well-developed root systems [45,46,47]. *Lespedeza davurica* is a leguminous species with a taproot system, while *S. bungeana* is a bunch grass with a fibrous root system [44]. In the present study, we investigated whether *L. davurica* and *S. bungeana* with contrasting root system exhibit different root trait plasticity and AM fungal responsiveness as a response to drought. To achieve this, we collected soils from a semiarid grassland of Northwestern China. We planted *L. davurica* and *S. bungeana* in monocultures in sterilized and live soils in a glasshouse, followed by a drought treatment. We then evaluated plant performance (i.e., plant biomass), root traits (e.g., root biomass allocation, SRL, RD, and root N and P content), root colonization by AM fungi, and soil nutrient availability. Specifically, we tested the following two hypotheses: (ⅰ) Fibrous-rooted *S. bungeana* develops more conservative root traits (e.g., thicker RD and lower SRL) than tap-rooted *L. davurica* as a response to drought; (ⅱ) Tap-rooted *L. davurica* depend more on AM fungi for water and nutrient acquisition than fibrous-rooted *S. bungeana* under drought conditions.

## 2. Results

### 2.1. Plant Biomass and Biomass Allocation

**Figure 1:**
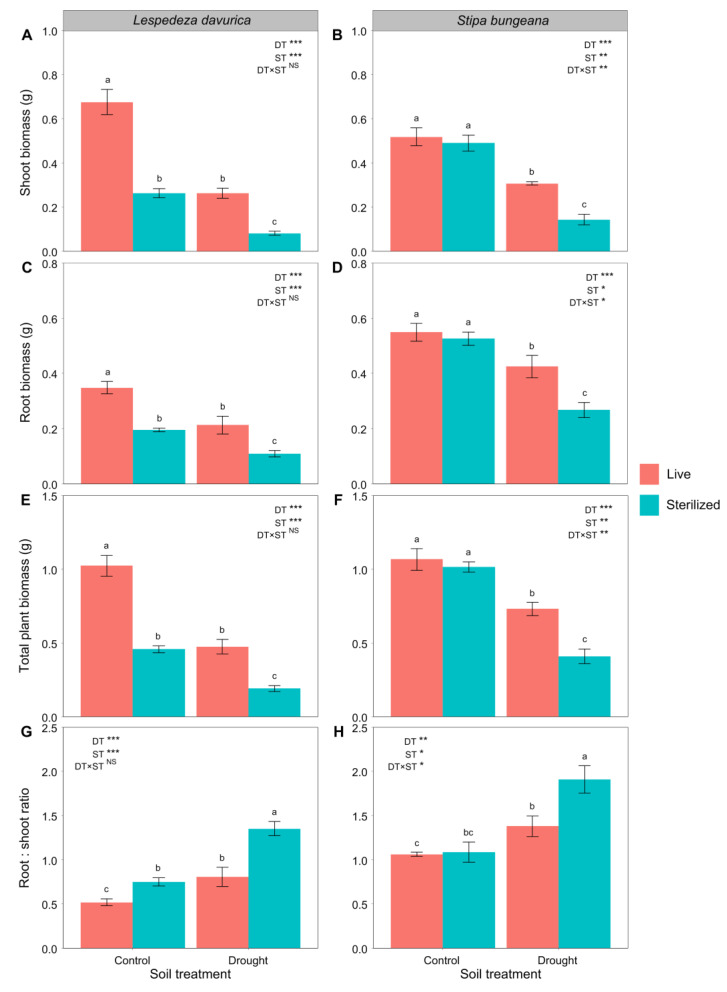
Shoot biomass, root biomass, total plant biomass, and the root:shoot biomass ratio after *Lespedeza davurica* (**A**,**C**,**E**,**G**) and *Stipa bungeana* (**B**,**D**,**F**,**H**) were grown in monocultures in live and sterilized soil under control or drought conditions. Results of two-way ANOVA analysis of the effects of drought treatment (DT), soil sterilization (ST), and their interactions are shown. * *p* < 0.05; ** *p* < 0.01; *** *p* < 0.001; NS, non-significant. Bars represent mean ± SE. Within each panel, different lowercase letters indicate significant differences between treatments at *p* < 0.05 (Tukey’s HSD).

Drought consistently decreased shoot biomass, root biomass, and total biomass but increased the root:shoot ratios (RSR) of *L. davurica* and *S. bungeana* (Table 1, Figure 1). However, soil sterilization had contrasting effects on plant performance in the two species under control and drought conditions (Table 1, Figure 1). For *L. davurica*, soil sterilization significantly decreased shoot biomass, root biomass, and total biomass and increased the RSR under both control and drought conditions (Figure 1A,C,E,G). In contrast, only under drought condition, soil sterilization significantly decreased shoot biomass, root biomass, and total biomass of *S. bungeana* and increased its RSR (significant drought treatment × soil sterilization treatment interaction; Table 1, Figure 1B,D,F,H).

### 2.2. Root Traits

**Figure 2:**
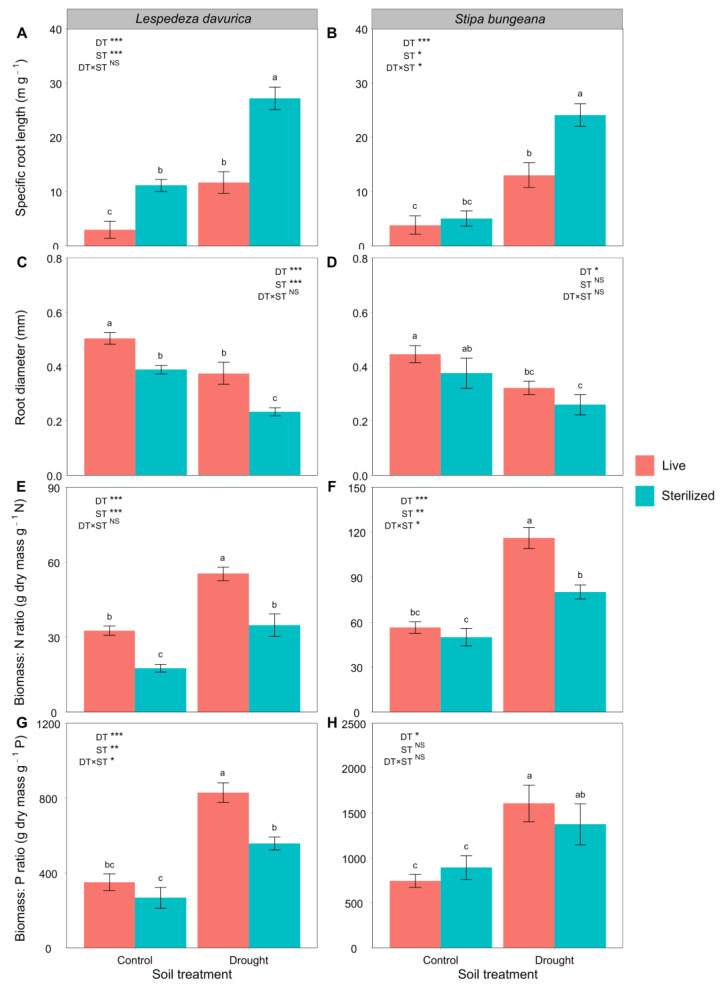
Specific root length, root diameter, and the biomass:N and biomass:P ratios after *Lespedeza davurica* (**A**,**C**,**E**,**G**) and *Stipa bungeana* (**B**,**D**,**F**,**H**) were grown in monocultures in live and sterilized soil under control or drought conditions. Results of two-way ANOVA analysis of the effects of drought treatment (DT), soil sterilization (ST), and their interactions are shown. * *p* < 0.05; ** *p* < 0.01; *** *p* < 0.001; NS, non-significant. Bars represent mean ± SE. Within each panel, different lowercase letters indicate significant differences between treatments at *p* < 0.05 (Tukey’s HSD).

Similarly, drought consistently increased SRL, biomass:N ratio, and biomass:P ratio but decreased RD of *L. davurica* and *S. bungeana* (Table 1, Figure 2). Under both control and drought conditions, soil sterilization significantly increased SRL in *L. davurica* but decreased its RD and biomass:N ratio (Figure 2A,C,E), but only under drought condition, it decreased biomass:P ratio (significant drought treatment × soil sterilization treatment interaction; Table 1, Figure 2G). Under drought conditions, soil sterilization significantly increased the SRL of *S. bungeana* but decreased its biomass:N ratio (significant drought treatment × soil sterilization treatment interactions; Table 1, Figure 2B,F). Soil sterilization had no significant effect on RD and biomass:P ratio of *S. bungeana* (Figure 2D,H).

### 2.3. Soil Nutrients

**Figure 3:**
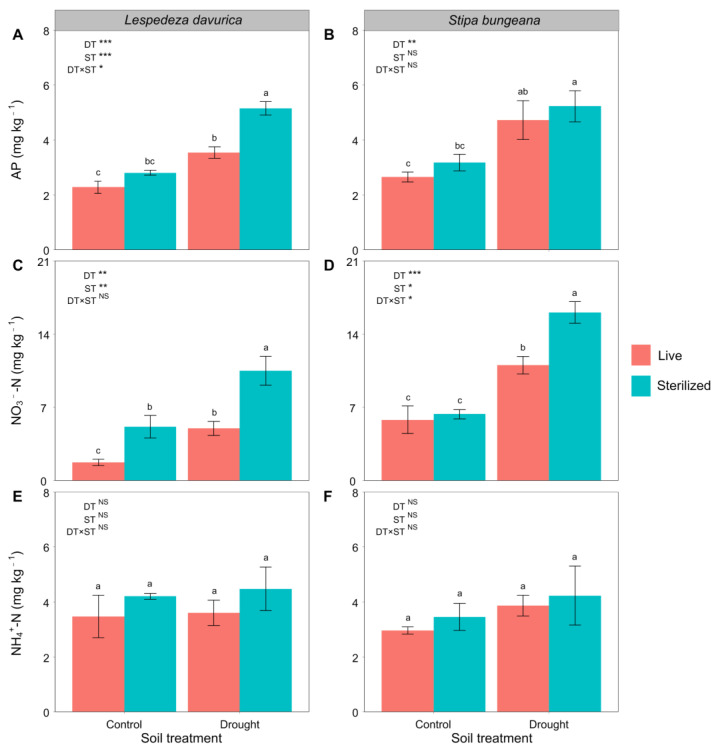
Soil available phosphorus (AP), NO_3_^−^-N, and NH_4_^+^-N after *Lespedeza davurica* (**A**,**C**,**E**) and *Stipa bungeana* (**B**,**D**,**F**) were grown in monocultures in live and sterilized soil under control or drought conditions. Results of two-way ANOVA analysis of the effects of drought treatment (DT), soil sterilization (ST), and their interactions are shown. * *p* < 0.05; ** *p* < 0.01; *** *p* < 0.001; NS, non-significant. Bars represent mean ± SE. Within each panel, different lowercase letters indicate significant differences between treatments at *p* < 0.05 (Tukey’s HSD).

At the end of the glasshouse experiment, drought significantly increased the concentration of available P and NO_3_^−^-N in the soils used to grow *L. davurica* and *S. bungeana* (Figure 3A–D). Only under drought condition, soil sterilization significantly increased the concentration of available P in the soil inhabited by *L. davurica* (significant drought treatment × soil sterilization treatment interaction; Table 1, Figure 3A). In contrast, soil sterilization had no significant effects on available P with *S. bungeana* under drought and control conditions (Figure 3B). Soil sterilization significantly increased the concentration of NO_3_^-^-N under control and drought conditions for *L. davurica* (Figure 3C) but only under drought conditions for *S. bungeana* (significant drought treatment × soil sterilization treatment interaction; Table 1, Figure 3D). Neither drought nor sterilization treatments had significant effects on the concentrations of NH_4_^+^-N in the soils used to grow *L. davurica* and *S. bungeana* (Figure 3E,F).

### 2.4. AM Fungal Colonization

**Figure 4:**
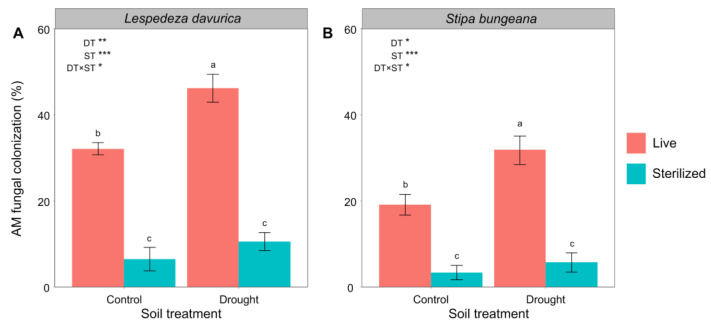
Arbuscular mycorrhizal (AM) fungal colonization in roots after *Lespedeza davurica* (**A**) and *Stipa bungeana* (**B**) were grown in monocultures in live and sterilized soil under control or drought conditions. Results of two-way ANOVA analysis of the effects of drought treatment (DT), soil sterilization (ST), and their interactions are shown. * *p* < 0.05; ** *p* < 0.01; *** *p* < 0.001. Bars represent mean ± SE. Within each panel, different lowercase letters indicate significant differences between treatments at *p* < 0.05 (Tukey’s HSD).

The AM fungal colonization on the roots of *L. davurica* and *S. bungeana* showed a consistent pattern in response to drought and soil sterilization (Table 1, Figure 4). Soil sterilization significantly reduced AM fungal colonization of both plant species (Figure 4A,B). Only in live soil, drought significantly increased AM fungal colonization of *L. davurica* and *S. bungeana* roots (significant drought treatment × soil sterilization treatment interaction; Table 1, Figure 4A,B).

## 3. Discussion

Understanding root trait plasticity responses to changing water availability is important in integrating root function into predictive models of soil resource use and plant performance, particularly under ongoing global climate change. In this study, we compared whether two grassland species with contrasting root system (i.e., taproot vs. fibrous-root) exhibit different root trait plasticity and associated AM fungal colonization as a response to drought. Contrary to our first hypothesis, we found that fibrous-rooted *S. bungeana* and tap-rooted *L. davurica* displayed the same pattern in biomass production and root trait variation (i.e., increased SRL and decreased RD) as a response to drought. Drought caused reductions in shoot biomass and root biomass, with more biomass allocation to roots of both species under drought conditions. This trend aligns with many previous studies documenting increased root biomass allocation under water-stressed environments [48,49,50] and is in support of the idea that plants would allocate more biomass to the organs involved in the absorption of the limiting resources [51,52,53,54].

In accordance with several previous studies [49,55], our study showed that both fibrous-rooted *S. bungeana* and tap-rooted *L. davurica* showed a significant increase in SRL but a decrease in RD, supporting the functional equilibrium hypothesis that plants are more acquisitive under conditions of lower resource availability [56,57,58]. Due to the limited absorption capacity for water and nutrients, shifts to thinner RD and higher SRL can promote root extension ability in dry soils that increases root absorptive area to maximize soil exploration and acquisition of limiting resources [19,59,60]. However, an overall decrease in SRL and increase in RD have also been reported [14,61], characterizing an alternative strategy in which plants may minimize plant water loss through lower specific hydraulic conductivity and lead to increased root longevity [62,63].

Another important finding was that, for *L. davurica*, soil sterilization significantly decreased total biomass and increased RSR and SRL under both control and drought conditions. However, only under drought condition, soil sterilization significantly decreased biomass of *S. bungeana* and increased its RSR and SRL. Using the approach of sterilization allowed for testing the net biotic effects contributing to the plant performance. Furthermore, by recording AM fungal colonization of the roots, we could detect the role that AM fungi played in the nutrient absorption and, thus, plant performance. We found that sterilization significantly decreased biomass of *L. davurica* and *S. bungeana*, which paralleled the responses of AM fungal colonization to sterilization. Arbuscular mycorrhizal fungi are increasingly considered extensions of roots, playing a vital role in helping plants forage soil resources [36,64,65,66,67]. The reduction in AM fungal colonization may decrease nutrient transportation in the soil [38,68], resulting in an increase in the concentrations of inorganic N (particularly NO_3_^−^-N) and available P in sterilized soil as observed in our study. However, in water-abundant conditions, biomass production and root trait plasticity of fibrous-rooted *S. bungeana* were similar between sterilized and live soil, which contrasts with the response of AM fungal colonization to sterilization. In line with many previous studies, we observed AM fungal colonization on roots of *L. davurica* and *S. bungeana* to significantly increase in response to drought. In addition, the fibrous-rooted *S. bungeana* and tap-rooted *L. davurica* had higher biomass:N and biomass:P ratios under drought conditions, suggesting the increased plant N and P use efficiency under drought conditions. These findings suggest that, in water-abundant conditions, tap-rooted *L. davurica* may depend more on AM fungi than fibrous-rooted *S. bungeana*; however, under drought conditions, AM fungi are of equal importance in favoring both plant species to forage soil resources independent of the variation in root system, therefore, partly supporting our second hypothesis.

Interestingly, we found that both fibrous-rooted *S. bungeana* and tap-rooted *L. davurica* not only became more acquisitive (higher SRL and lower RD) but also had higher AM fungal colonization under drought conditions, implying a complementarity in water and nutrients foraging between the roots and the associated AM fungi [42,69,70,71,72]. This complementarity could be a bet-hedging strategy that plants use to forage soil resources under stressed environments. Although thinner RD and higher SRL can promote root extension ability in dry soils, investment in AM fungi may be more efficient in foraging the limiting resources [37,73,74,75,76]. This is because AM fungi can either act as hollow tubes that transport water directly from soil pores to the root tissues [77,78] or alter plant water balance indirectly by modifying the hormonal profiles or improve plant nutritional status [37,79,80]. Our findings suggest that roots and AM fungi are complementary in foraging sources and, thus, provide new insights into water and nutrient absorption for fibrous-rooted species that may shift from root-driven under water-abundant conditions to mycorrhizae-driven under water-stressed environments.

While a consistent variation towards resource-acquisitive root traits (increased RSR and SRL and decreased RD) were observed in the fibrous-rooted *S. bungeana* and tap-rooted *L. davurica* as a response to drought, previous studies also reported contrasting findings where drought decreased SRL and increased RD [14,61]. Therefore, a wider spectrum of plant species, including woody and herbaceous species, would be needed to test the generality of the pattern across the arid and semiarid areas. In addition, the whole-soil inoculum used in our study contained complex communities of soil organisms. Consequently, the net positive effects of soil sterilization on plant performance are not limited to only AM fungi but can be attributed to interactions between plants and many other soil organisms. For instance, dark septate endophytes (DSE) might play a key role in the two species under drought conditions. The DSE might serve a similar role as mycorrhizal fungi favoring plant performance with more colonization in water-stressed and nutrient-limited environments, such as arid and semiarid areas [81,82,83]. Moreover, plant growth-promoting rhizobacteria (PGPR) are natural rhizosphere-inhabiting bacteria [84,85,86], which also facilitates plant growth and development by contributing to enhanced nutrient acquisition by host plants, thus, protecting against phytopathogenic microbes and promoting resistance to various abiotic stresses [87,88]. The effects of PGPR on plant growth can be exerted by mechanisms including secretion of plant growth-regulating substances, such as auxins, cytokinins, and bacterial volatiles [89,90,91,92]. Furthermore, drought is expected to alter plant physiology and metabolic pathways [93], and plants are likely to alter the level of their hormones to adapt to resource-limited environments. For instance, salicylic acid and indole-3-acetic acid interact with jasmonic acid, thus, regulating the adaptation of plants to their surroundings [94]. Therefore, the physiological regulatory mechanisms should be considered when detecting the roles of beneficial microbes in facilitating plant growth under drought stress.

## 4. Materials and Methods

### 4.1. Study Site

The study site was located at the Grassland Research Station of Lanzhou University (LZUGRS), Huan County, Gansu Province, Northwestern China (37.12° N, 106.82° E, 1650 m a.s.l.). The area has a typical semiarid monsoon climate, with a mean annual temperature and rainfall of approximately 8.5 °C and 360 mm, respectively, and more than 60–80% of which occurs from June to September. It has cambisol [95] soil type dominated by forb *Artemisia capillaris* Thunberg (Asteraceae), semi-shrub *Lespedeza davurica* (Laxm.) Schindl (Fabaceae), and bunch grass *Stipa bungeana* Trin (Poaceae) [44,96].

### 4.2. Study Plants and Seed Collection

We selected two dominant perennial plant species (*L. davurica* and *S. bungeana*) with contrasting root system architectures as the study plants (for basic parameters of the two species see Table 2). *Lespedeza davurica* is a leguminous species with a taproot system consisting of a taproot and coarse lateral roots. The taproot is developed first from the radical becoming the most prominent root, followed by the emergence of many smaller roots branching from the taproot. *Stipa bungeana* is a bunch grass with a fibrous root system and numerous same-sized fine roots developing from the radical [44]. The seeds of the two species were harvested from the same population growing on the grasslands 1–2 km from LZUGRS in 2018. The harvested seeds were air-dried, cleaned, and stored at 4 °C in the laboratory.

### 4.3. Soil Sampling and Processing

Soil samples were collected along a Z-shaped transect with about 10 m distance between adjacent sampling positions using a shovel from the grasslands 1–2 km from LZUGRS on 8 May 2019. All soil samples were sieved through a 5 mm mesh to remove large roots and plant residues and, then, were bulked and homogenized to create a composite sample. The homogenized soil was divided into two parts. A small fraction of the soil was used as a live soil inoculum. The remaining soil was sterilized using γ-radiation (≥25.0 kGy) at Tianchen Radiation Co., Ltd., Lanzhou, China, and stored at 4 °C, awaiting use as the background soil in the glasshouse experiment.

### 4.4. Glasshouse Experiment

**Figure 5:**
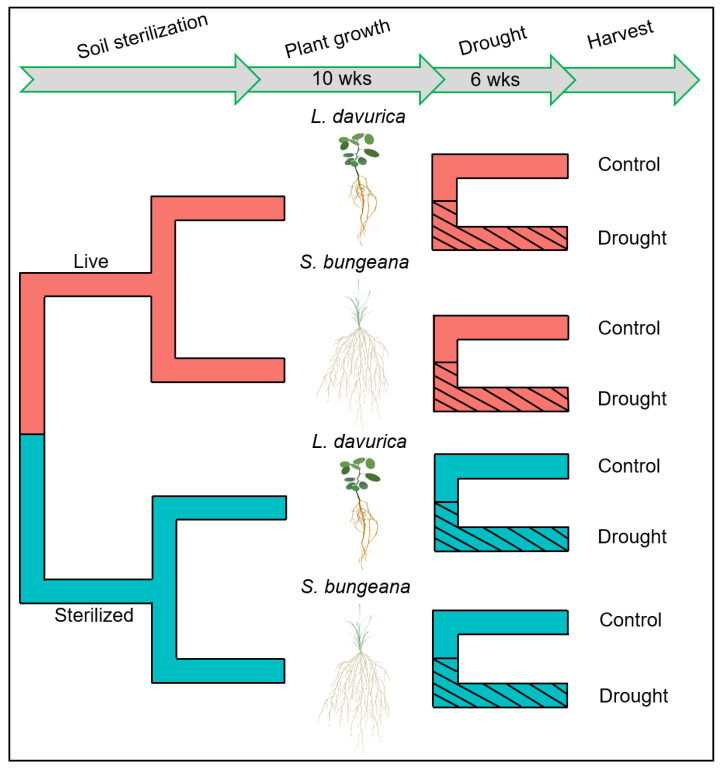
Schematic depiction of the experimental design. Tap-rooted *Lespedeza davurica* and fibrous-rooted *Stipa bungeana* from a semiarid grassland in Northwest China were grown in monocultures in live and sterilized soils in a glasshouse, followed by a drought treatment. The live and sterilized soils were obtained by inoculating a small amount of live or sterilized soil to sterilized background soil in a mass ratio of 1:10. After 10 weeks, half of the pots of each species were well-watered and half were subjected to drought conditions for 6 weeks.

To test whether tap-rooted *L. davurica* and fibrous-rooted *S. bungeana* exhibit different root trait plasticity and AM fungal responsiveness as a response to drought, we grew them in monocultures in plastic pots (15 cm diameter × 20 cm deep) containing live or sterilized soils, followed by a drought treatment. There were eight treatments (2 plant species × 2 soil sterilization treatments × 2 drought treatments) (Figure 5). Each treatment was replicated 15 times, resulting in 120 pots. To minimize the possible side effects of nutrient release caused by sterilization, a small amount of live and sterilized soil (10% mass) was added to the background soil to obtain the ‘live’ or ‘sterilized’ soils, respectively [97]. Before the glasshouse experiment, the chemical properties of the ‘sterilized’ and ‘live’ soil were analyzed. The concentrations of soil organic C, total N, total P, available P, and inorganic N (NO_3_^−^-N and NH_4_^+^-N) did not differ significantly between sterilized and live soil (methods see below; results see Table 3), indicating that the approach of adding a small amount of live soil did not alter the nutrient availability of the treatment-specific sterilized background soil.

Seeds of *L. davurica* and *S. bungeana* were surface-sterilized using 70% ethanol for 1 min and 1% NaClO for 2 min, followed by rinsing three times with sterile distilled water. The sterile seeds were germinated in sterilized vermiculite at room temperature in the laboratory. On 10 June 2019, one-week-old seedlings (five per pot) were transplanted into plastic pots containing live or sterilized soils in monocultures. The bottom of each pot was filled with 200 g of sterilized sand, followed by 1500 g of sterilized background soil mixed with either 150 g of sterilized or live soil inoculum, and topped off with 200 g of sterilized sand. The pots were randomly arranged in a glasshouse maintained at 60% relative humidity, 16/8 h day/night cycle, and 21/16 °C day/night temperature. Seedlings that died during the first week after transplanting were replaced immediately. Pots were re-arranged weekly to avoid possible positioning effects. Seedlings were watered as necessary with tap water for ten weeks before the onset of the drought treatment. On 22 August 2019, we started drought treatment: half the pots were well-watered every other day with 400 g water, maintaining them at 60% water-holding capacity (WHC), and the other half were watered with 130 g water by maintaining them at 20% WHC and served as drought condition for six weeks. On 3 October 2019, all the plants were harvested, and soil samples were collected and stored at 4 °C for nutrient analyses. A subset of the roots was weighed and stored in 50% ethanol at 4 °C for the assessment of AM fungal colonization.

### 4.5. Plant Measurements

Aboveground biomass on each pot was clipped at the soil surface, dried at 70 °C for 48 h, and weighed. Roots were carefully removed from the soil and washed. The root surface area, volume, length, and diameter were then determined using the WinRHIZO Pro 2019a root analysis software (Regent Instruments, Sainte Foy, QC, Canada). The rest of the roots were dried at 70 °C for 48 h and weighed. The dried shoots and roots were ground using the Retsch Ball Mill MM 400 (Retsch, Dusseldorf, Germany), and the N and P concentrations in the shoots and roots were determined using a Smartchem 450 Discrete Auto Analyzer (AMS, Rome, Italy). Next, the dry root-to-shoot biomass ratios (RSR) were calculated. The SRL was determined as the root length to root dry mass ratio [98]. The whole-plant biomass to N content (g dry mass g^−1^ N) and P content (g dry mass g^−1^ P) ratios were assessed as a proxy of N and P use efficiency, respectively [99].

### 4.6. Soil Measurements

The soil organic C (SOC), total N, total P, available P (AP), and inorganic N (NO_3_^−^-N and NH_4_^+^-N) concentrations in sterilized and live soil samples were analyzed before and after the greenhouse experiment. The soil total N and total P concentrations were determined by adding 1.65 g of catalyst (K_2_SO_4_ vs. CuSO_4_ at a ratio of 10:1) and 5 mL of concentrated sulfuric acid to a 0.5 g soil sample, then maintaining it for 1.5 h at 420 °C for digestion, and finally analyzed with a Smartchem 450 Discrete Auto Analyzer (AMS, Rome, Italy). The SOC was determined using the Walkley–Black method [100]. The AP was determined using the 0.5 M NaHCO_3_ extraction-molybdenum-antimony anti-spectrophotometric method [101]. Inorganic N (NO_3_^−^-N and NH_4_^+^-N) was extracted from a 5 g soil subsample using 25 mL of 1 M KCl. The extracts were passed through a Whatman No. 1 filter paper, then subjected to a colorimetric analysis with a Smartchem 450 Discrete Auto Analyzer (AMS, Rome, Italy).

### 4.7. Arbuscular Mycorrhizal Fungi in Roots

Root samples were cleared in 10% potassium hydroxide for 30 min at 90 °C and stained with acidic glycerol solution containing 0.05% trypan blue [102]. The AM fungi were distinguished as arbuscules, vesicles, and hyphae and the AM fungal colonization was determined using the gridline intersect method at 200× magnification [103].

### 4.8. Statistical Analyses

All statistical analyses were performed using R v. 3.5.1 [104]. All plant and soil data were analyzed using two-way ANOVA, with soil sterilization, drought treatment, and their interactions as fixed factors. Whenever significant interactions were detected, post hoc tests were performed using the ‘multcomp’ package in R with Tukey HSD adjustment, which accounts for multiple comparisons. Model assumptions were checked using the Shapiro–Wilk test of normality and the Levene’s test of heterogeneity of variance. All data were transformed as necessary to meet the model assumptions (see Table 1 for details).

## 5. Conclusions

Our results reveal that fibrous-rooted *S. bungeana* and tap-rooted *L. davurica* have the same root trait variation pattern characterized by increased RSR and SRL and decreased RD under drought conditions. However, for *L. davurica*, soil sterilization increased RSR and SRL under both control and drought conditions, whereas only under drought condition, soil sterilization increased RSR and SRL of *S. bungeana*. Sterilization significantly decreased biomass of *L. davurica* and *S. bungeana*, which paralleled the responses of AM fungal colonization to sterilization. Our findings suggest that, in water-abundant conditions, tap-rooted *L. davurica* may depend more on AM fungi than fibrous-rooted *S. bungeana*; however, under drought conditions, AM fungi are of equal importance in favoring both plant species to forage soil resources. Our study indicates that roots and AM fungi are complementary in foraging sources under water-stressed environments. These findings provide important information for understanding the resource utilization strategies and performance of plant species in arid and semiarid grasslands in the ongoing climate change.

## Figures and Tables

**Table 1:** Results from two-way ANOVA analysis of the effects of drought treatment, soil sterilization, and their interactions on shoot biomass, root biomass, total plant biomass, root:shoot biomass ratio, specific root length, root diameter, biomass:N ratio, biomass:P ratio, soil available P, inorganic N (NO_3_^−^-N and NH_4_^+^-N), and AM fungal colonization after *Lespedeza davurica* and *Stipa bungeana* were grown in monocultures in the glasshouse.

Variables	*Lespedeza davurica*			*Stipa bungeana*		
	Drought Treatment (DT)	Soil Sterilization (ST)	DT × ST	Drought Treatment (DT)	Soil Sterilization (ST)	DT × ST
Shoot biomass †	141.60 ***	141.60 ***	1.71	83.93 ***	19.13 **	14.45 **
Root biomass †	26.06 ***	32.04 ***	0.11	34.21 ***	9.75 *	6.78 *
Total plant biomass †	89.45 ***	95.98 ***	0.39	69.00 ***	16.55 **	12.10 **
Root:shoot ratio	36.26 ***	27.46 ***	4.49	25.24 **	5.98 *	5.95 *
Specific root length	50.69 ***	46.44 ***	4.44	56.63 ***	10.69 *	6.88 *
Root diameter	31.26 ***	25.46 ***	0.29	9.49 *	2.89	0.01
Biomass:N ratio	47.91 ***	37.93 ***	0.77	64.61 ***	15.20 **	6.96 *
Biomass:P ratio	66.92 ***	14.45 **	6.15 *	5.48 *	0.02	0.43
Available P	86.55 ***	30.18 ***	7.55 *	19.23 **	1.22	0.00
NO_3_^−^-N	20.68 **	22.31 **	1.23	59.71 ***	8.96 *	5.37 *
NH_4_^+^-N	0.11	1.78	0.01	1.80	0.46	0.01
AM fungal colonization	13.70 **	155.97 ***	5.15 *	10.09 *	76.29 ***	5.85 *

Data are represented as F-values and asterisks indicates significant *p*-values (* *p* < 0.05; ** *p* < 0.01; *** *p* < 0.001); † Data log(x)-transformed before analysis.

**Table 2:** The basic parameters of *Lespedeza davurica* and *Stipa bungeana* used in the study.

Plant Species	Family	Category	Life History	Cotyledon Type	Root System Type	Mycorrhizal Dependence
*Lespedeza davurica*	Leguminosae	Semi-shrub	Perennial	Bicotyledon	Tap-root	Yes
*Stipa bungeana*	Gramineae	Bunch grass	Perennial	Monocotyledon	Fibrous root	Yes

**Table 3:** The concentrations of soil organic C, total N, total P, available P, and inorganic N (NO_3_^−^-N and NH_4_^+^-N) of soil followed by sterilized and unsterilized treatment before growing plants in the glasshouse experiment.

Soil Treatment	Soil Organic C (g/kg)	Total N (g/kg)	Total P (g/kg)	Available P (mg/kg)	NO_3_^−^-N (mg/kg)	NH_4_^+^-N (mg/kg)
Unsterilized	4.96 (0.29)	0.64 (0.04)	0.53 (0.01)	3.14 (0.17)	2.38 (0.02)	11.54 (0.07)
Sterilized	5.36 (0.45)	0.67 (0.05)	0.50 (0.05)	3.58 (0.09)	2.56 (0.05)	10.59 (0.14)

Data are represented as the mean with standard errors in brackets (*n* = 6).

## Data Availability

All the data used in the study are within the manuscript.
